# Proinflammation, profibrosis, and arterial aging

**DOI:** 10.1002/agm2.12099

**Published:** 2020-03-18

**Authors:** Mingyi Wang, Robert E. Monticone, Kimberly R. McGraw

**Affiliations:** ^1^ Laboratory of Cardiovascular Science National Institute on Aging National Institutes of Health Baltimore Maryland

**Keywords:** aging, artery, collagen, profibrosis, proinflammation, stiffening

## Abstract

Aging is a major risk factor for quintessential cardiovascular diseases, which are closely related to arterial proinflammation. The age‐related alterations of the amount, distribution, and properties of the collagen fibers, such as cross‐links and degradation in the arterial wall, are the major sequelae of proinflammation. In the aging arterial wall, collagen types I, II, and III are predominant,  and are mainly produced by stiffened vascular smooth muscle cells (VSMCs) governed by proinflammatory signaling, leading to profibrosis. Profibrosis is regulated by an increase in the proinflammatory molecules angiotensin II, milk fat globule‐EGF‐VIII, and transforming growth factor‐beta 1 (TGF‐β1) signaling and a decrease in the vasorin signaling cascade. The release of these proinflammatory factors triggers the activation of matrix metalloproteinase type II (MMP‐2) and activates profibrogenic TGF‐β1 signaling, contributing to profibrosis. The age‐associated increase in activated MMP‐2 cleaves latent TGF‐β and subsequently increases TGF‐β1 activity leading to collagen deposition in the arterial wall. Furthermore, a blockade of the proinflammatory signaling pathway alleviates the fibrogenic signaling, reduces profibrosis, and prevents arterial stiffening with aging. Thus, age‐associated proinflammatory‐profibrosis coupling is the underlying molecular mechanism of arterial stiffening with advancing age.

## 1 INTRODUCTION

Aging is a major risk factor for the morbidity and mortality of quintessential cardiovascular diseases, such as hypertension and atherosclerosis, mainly due to arterial wall structural and functional adverse remodeling, such as intimal medial thickening (IMT) and stiffening.^1^, ^2^, ^3^, ^4^, ^5^ The age‐associated increase in collagen deposition within the arterial wall is known as arterial profibrosis; and the age‐associated increase in sterile inflammation within the wall is known as arterial proinflammation. Proinflammation and profibrosis are the key molecular and cellular events in age‐associated IMT and arterial stiffening. It is widely accepted that proinflammatory endothelial cells (ECs) and vascular smooth muscle cells (VSMCs) are mainly responsible for age‐associated adverse arterial cellular events; however, the consequence of proinflammation and profibrosis (predisposing collagen deposition) greatly affects the behavior of these cells adversely with a predominant impact on the age‐associated arterial stiffening, which is not completely understood.^1^, ^2^, ^3^, ^4^, ^5^

Aging increases the proinflammatory molecules angiotensin II (Ang II), milk fat globule‐EGF8 (MFG‐E8), calpain‐1, monocyte chemoattractant protein 1 (MCP‐1), non‐phagocytic nicotinamide adenine dinucleotide phosphate oxidase (NADPH oxidase), and endothelin‐1 (ET‐1).^1^, ^2^, ^3^, ^4^, ^5^ Proinflammation activates transforming growth factor‐beta 1 (TGF‐β1), a profibrogenic signal and also triggers the activation of matrix metalloproteinase type II (MMP‐2) in VSMCs.^5^, ^6^ Activated MMP‐2 increases collagen production, via profibrogenic signaling, and degeneration, contributing to EC and VSMC dysfunction.^5^, ^6^ Conversely, aging decreases the extracellular anti‐inflammatory molecule vasorin, which also contributes to the activation of MMP‐2, TGF‐β1 profibrogenic signaling, and collagen secretion of VSMCs.^7^, ^8^

Profibrotic collagen deposition and cross‐linked modification execute a causal role providing cellular signals that regulate the degenerative phenotype of ECs and the migration, invasion, proliferation, and proinflammatory gene expression, and stiffening of VSMCs in vitro.^1^, ^2^, ^3^, ^4^, ^9^ Collagen deposition and cross‐linking also play etiologic roles in IMT, endothelial dysfunction, and stiffening in vivo,^1^, ^2^, ^3^, ^4^, ^9^, ^10^, ^11^ facilitating the development of hypertension and atherosclerosis with aging.^1^, ^2^, ^3^, ^4^, ^9^

This review focuses on the molecular components of the arterial proinflammatory profibrogenic signaling cascade in the cells of the arterial walls with advancing age.

## 2 COLLAGEN TYPES AND THEIR ROLES IN THE ARTERIAL WALL

Individual collagen polypeptide chains contain many repeat amino acid sequences, most often Gly‐Xaa‐Yaa, where Xaa is often Proline (Pro) and Yaa is often 4‐hydroxy‐l‐proline. All types of collagen have a characteristic triple‐helical structure in which the three helical chains are staggered by one residue and are supercoiled in a right‐handed manner. Appropriate folding of collagen into its triple helix followed by processing of N‐ and C‐propeptide is critical for the formation and stability of the extracellular matrix.

Collagen is the most abundant protein in the body compromising 25%‐30% of the total protein.^12^ Generally, 90% of collagens in the body are collagen types I and III.^13^ Type I and III collagens account for 60% of the artery.^14^ Collagen I is composed of one alpha1 (I) and two alpha2 (I) chains that are twisted into a mature collagen fiber. Collagen II is composed of the three‐identical alpha 1(II) chains twisted into the mature type II collagen fiber. Collagen III is also composed of the three identical alpha 1 (III) chains twisted into the mature type III collagen fiber. Collagen fibers are 100‐1000 times stiffer than mature elastin fibers, which causes a dramatic increase in the incremental elastic modulus (stiffness) of the arterial wall at higher levels of circumferential stretch.^15^

In general, collagen type I appears thicker, and converts the tensile strength (stiffening) to the arterial wall.^16^, ^17^, ^18^ The increase in collagen type I is related to the stiffening of the arterial wall. Collagen type II is an element of cartilage that is rarely detected in young arterial walls but is often found in the old arterial wall and is linked to age‐associated arterial procalcification.^19^ Collagen type III appears thinner and more elastic and exerts resilience.^20^, ^21^ The increase in collagen type III is related to arterial adaptive remodeling.^22^

## 3 PROFIBROSIS IN THE AGING ARTERIAL WALL

Collagen in the arterial wall is produced mainly by VSMCs and its homeostasis is important to arterial health. The age‐associated increase in collagen deposition within the arterial wall is known as arterial profibrosis. In the aging aortic wall of rats, the amount of total collagen increases in older animals (30 vs 8 months): collagen type I increases while collagen type III decreases, particularly in the thickened intima.^23^ Importantly, the ratio of collagen/elastin increases with age.^23^ Similarly, in the coronary arterial wall of aging rats, total collagen fraction increases, and collagen III decreases while collagen type I increases.^24^ Increases in collagen deposition have been observed in aortic walls from post‐mortem analysis of aortae taken from normotensive subjects aged from 14 to 90 years.^25^ The concentration of thoracic aortic collagen significantly increases by 72% in old versus young normotensive subjects.^25^ In aged grossly normal human aortae, collagen types I and III markedly increase within the arterial wall.^26^ Interestingly, collagen type I cleaved ¼‐length or ¾‐length fragments also increase in older arterial walls, suggesting that increases in collagen deposition and collagen degeneration co‐exist during aging.^6^ Notably, the fragments serve as molecular cues and effectively affect the phenotypes of ECs or VSMCs.^27^, ^28^

## 4 COLLAGEN PRODUCTION IN AGING VSMCS

In the arterial wall, collagen has a half‐life of 60‐70 days.^29^ The activity of myofibroblasts or osteoblast‐like transdifferentiated VSMCs populating the arterial wall creates a continuous turnover of collagen that is the biological basis of arterial structure conservation.^30^

### 4.1 Collagen expression

Aged VSMCs are often exposed to various proinflammatory factors, such as Ang II, leading to the activation of TGF‐β1 and connective tissue growth factor (CTGF) expression that facilitate p‐SMAD2/3 and ETS‐1 translocation into nuclei; these transcription factors activate the promoter for collagen genes and increase collagen I, II, and III mRNA in the arterial wall.^19^, ^31^, ^32^ In addition, collagen types I and III also increased in age‐associated metabolically remodeled aortic walls in nonhuman primates.^33^

### 4.2 Collagen maturation

The maturation of procollagen type I is a multistep process that requires the participation of several enzymes and chaperone molecules to ensure the faithful secretion of this trimeric protein in VSMCs.^34^, ^35^ Within the endoplasmic reticulum (ER), two proα1 (I) collagen chains and one proα2 (I) collagen are initially joined at their globular carboxyl termini. This interaction is initiated by protein‐disulfide isomerase (PDI) and stabilized by the inter‐chain disulfide bonds; winding of the long triple helical domains then proceeds in the carboxyl to amino direction.^36^ The fidelity of this helix‐folding reaction is dependent on hydroxylation of proline residues by prolyl‐4‐hydroxylase (P4H).^37^

### 4.3 Collagen secretion

The process of collagen secretion by VSMCs is complex, involving the following molecular events. Protein kinase C subunit δ (PKCδ) is a critical mediator of collagen I secretion in VSMCs. Inhibition of PKCδ promotes the intracellular accumulation of procollagen I in the trans‐Golgi in VSMCs.^38^ Cell division control protein 42 homolog (CDC42) is reduced in PKCδ‐deficient VSMCs, contributing to a blockade of collagen I secretion.^38^ Restoration of PKCδ expression restores collagen I secretion and CDC42 expression, which also partially rescues collagen I secretion.^38^

Heat shock protein‐47 (HSP47) is identified as a collagen‐binding protein, residing in the ER, and functions as a collagen‐specific molecular chaperone. Once the triple helix is formed, the collagen is transported to the Golgi apparatus and brought to the cell surface after a period of Golgi cisternal maturation. HSP47 is expressed only by collagen‐producing cells and binds to collagen type I in vitro.^35^ HSP47 is present exclusively in the ER and plays a vital role in procollagen processing.^35^ Within the ER, HSP47 interacts with nascent type I procollagen chains with fully translated procollagen chains, with non‐helical and poorly hydroxylated, triple helical procollagen.^35^ Once the procollagen‐HSP47 complex reaches the cis‐Golgi, HSP47 dissociates and is recycled back to the ER, becoming involved in collagen secretion.^35^

### 4.4 Collagen modifications

Collagen fibrils are normally cross‐linked elegantly to optimize their strength and elasticity. Free amino groups on these collagen proteins, however, are susceptible to glycation and, eventually, advanced glycation end‐products (AGEs) form. The AGEs render the collagen fibrils resistant to normal hydrolytic turnover, thereby leading to a thicker and less distensible vascular matrix. AGEs are also a heterogeneous group and can further be modified via dehydration, condensation, fragmentation, cyclization, or oxidation reactions. Specificcaly, oxidation leads to more permanent, irreversible chemical modifications, such as glyco‐oxidation products, carboxymethyl lysine, and pentosidine, which have been used as surrogate markers for age‐related glycation of proteins.^39^ Modified collagen protein‐protein cross‐linking causes a loss of normal elasticity, strength, and flexibility.

The extracellular matrix (ECM) contains biologically cryptic sites that are exposed upon structural or conformational changes known as “matrikrines.”^40^, ^41^ For example, collagen contains multiple Arg‐Gly‐Asp (RGD) sites that do not appear to play an important role in integrin ligation of native collagen; however, cell attachment to denatured collagen was found to be mediated through these RGD motifs, suggesting that the denaturating process reveals “novel” biologically active sites.^42^ These “matricryptic” sites are now thought to be revealed by several stimuli, including enzymatic breakdown of the ECM, multimerization, mechanical stimuli, and denaturation or modification of the ECM.^40^, ^41^

## 5 PROINFLAMMATION PROMOTES PROFIBROSIS IN AGING ARTERIAL WALLS

**Figure 1:**
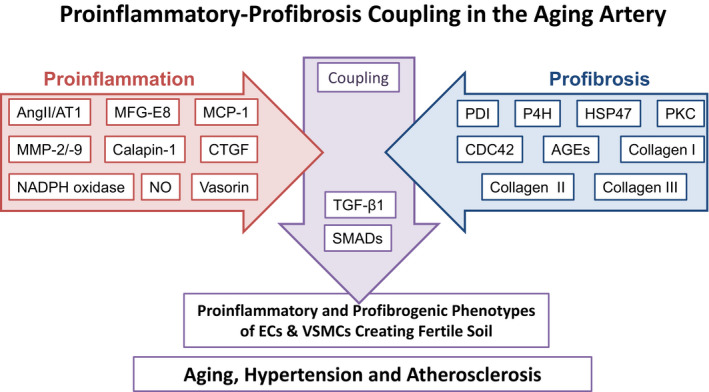
Diagram depicting the arterial proinflammatory‐profibrosis coupling events. AGEs, advanced glycosylation end‐products; AngII, angiotensin II; AT1, Ang II type 1 receptor; CDC42, cell division control protein 42 homolog; CTGF, connective tissue growth factor; ECs, endothelial cells; HSP47, heat shock protein 47; MCP‐1, monocyte chemoattractant protein‐1; MFG‐E8, milk fat globule epidermal growth factor‐8; MMPs, matrix metalloproteinases; NADPH oxidase, nicotinamide adenine dinucleotide phosphate oxidase; NO, nitric oxide; P4H, prolyl‐4‐hydroxylase; PDI, protein‐disulfide isomerase; PKC, protein kinase C; SMADs, homologies to the *Caenorhabditis elegans* SMA (“small” worm phenotype) and Drosophila MAD (“Mothers Against Decapentaplegic”) family of proteins; TGF‐β1, transforming growth factor‐beta 1; VSMCs, vascular smooth muscle cells.

A growing body of evidence indicates that Ang‐II‐associated proinflammatory molecules not only cause a low‐grade sterile inflammation but also serve as profibrogenic molecules promoting collagen production in VSMCs with aging (Figure 1).

### 5.1 Angiotensin II

The transcription, translation, and activity of angiotensin converting enzyme‐1 (ACE‐1) increases within the aging arterial wall, predominantly in the thickened intima in several species, including humans.^26^, ^31^, ^43^, ^44^ Additionally, chymase, an alternative angiotensin convertase, mainly derived from activated adventitial mast cells, has been detected only in the aging arterial wall and increases in nonhuman primates.^43^ Thus, Ang II, the cleaved product of angiotensinogen by these enzymes, is increased in the aged arterial wall, and in the thickened intima of rats, nonhuman primates, and particularly humans.^43^

In addition, the Ang II cognate receptor, AT1, is activated and upregulated within aged arterial walls.^26^ Type‐1 Ang II receptor‐associated protein (AGTRAP) is a molecule that specifically interacts with the carboxyl‐terminal domain of AT1R in VSMCs.^9^, ^45^, ^46^, ^47^ AGTRAP suppresses Ang II effects by promoting AT1R internalization or desensitization.^48^, ^49^ Importantly, aging decreases AGTRAP expression in the arterial wall, which inhibits the proliferation and senescence of VSMCs,^9^, ^46^ demonstrating that AGTRAP is a negative regulator of Ang II proinflammation.

### 5.2 Matrix metalloproteinase type II and transforming growth factor‐beta 1

Ang II activates MMP‐2 in aging VSMCs.^44^ MMP‐2, also known as gelatinase A, is a calcium‐dependent zinc‐containing endopeptidase. The aged intimal collagen secreted by cells is rapidly post‐modified via glycation and oxidation, rendering them resistant to cleavage by MMP‐2.^50^ Thus, an abundance of collagen can be detected in the thickened intima, which may contain abnormally increased levels of activated MMP‐2.^23^

Ang II stimulates TGF‐β1 activation through MMP‐2. TGF‐β1 plays multiple roles in arterial vascular remodeling, especially in arterial fibrosis.^51^ TGF‐β1 expression is temporospatially associated with an increase of collagen mRNA expression and local fibrosis in the sites of injured arteries, suggesting that TGF‐β1 plays a causal role in collagen deposition during arterial wall healing.^52^ With aging, large amounts of TGF‐β1 are present in ECs and in migrated intimal VSMC.^51^ The steady‐state mRNA for TGF‐β1 was found to increase in aging normotensive rats.^53^

In vitro studies show the ECs and VSMCs treated with TGF‐β1 increases mRNA levels of collagen types I and III, which are attenuated by a TβRII receptor blocker.^54^, ^55^ Li et al^56^ reported that an enhanced expression of TGF‐β1 in the thickened vascular intima of aged rats may be produced by an exaggerated VSMC response to cytokines, such as interleukin 6 (IL‐6), and may have potential roles in intimal remodeling with aging.

Notably, secreted TGF‐β1 is inactive since it is bound to latent TGF binding protein (LTBP)‐1 and latent associated protein (LAP).^51^ The latent TGF‐β1 is activated via the cleavage of these proteins by proteinase, eg, activated MMP‐2.^51^ Activated MMP‐2 in situ increases in the rat, monkey, and human aortae,^23^, ^26^, ^43^ and actives TGF‐β1 via the cleavage of LTBP‐1 and LAP in a step‐wise fashion.^51^ Thus, activated MMP‐2 increases collagen production in aging VSMCs.^51^

### 5.3 Monocyte chemoattractant‐1

MCP‐1/chemokine (C‐C notif) ligand2 (CCL2) is originally considered as one of the key chemokines that modulate migration and infiltration of monocytes/macrophages. Both MCP‐1 and its receptor CCR2 have been demonstrated to be induced and involved in various vascular diseases. MCP‐1 is regarded as a notorious inflammatory vascular factor. Both MCP‐1 and CCR2 mRNAs and proteins increased in old versus young F344xBN rat aortae.^57^ Cellular MCP‐1 and CCR2 are expressed by early‐passage VSMCs isolated from old aortae, which exhibit increased invasion capacity compared with young cells.^57^ Further, MCP‐1 treatment of young VSMCs induces migration and increases their ability to invade a synthetic basement membrane.^57^

Ang II increases the activation of MCP‐1,^58^ which increases MMP‐2 activity.^59^ The formation of an MCP‐1/MMP‐2/TGF‐β1 signaling loop increases collagen production in VSMCs with aging.^6^, ^59^

Altogether, Ang II, MCP‐1, CCR2, and collagen increase in the arterial wall and VSMCs with aging. MCP‐1 enhances VSMC migration, invasion, and collagen production. Ang II/MCP‐1/CCR2 signaling plays an inflammatory role in the induction of age‐associated arterial fibrotic remodeling.

### 5.4 Calpain‐1

Calpain‐1 is an intracellular calcium‐dependent proteinase that is increased by Ang II in aging VSMCs.^31^ Calpain‐1 activates MMP‐2,^19^ which signals VSMC collagen production via TGF‐β1.^51^ Activated MMPs also increases collagen fragmentation, which induces distinct integrin signals that lead to the initiation of calpain‐mediated cleavage of focal adhesion kinase (FAK), paxillin, talin adhesion molecules, and dissolution of the focal adhesion complex, altering the phenotype of VSMCs.^60^

### 5.5 Milk fat globule EGF‐VIII

MFG‐E8 is a milk fat protein that is increased in the aged rat, monkey, and human aortic wall. Ang II increases MFG‐E8, which increases MMP‐2 activation, TGF‐β1 activation and collagen production, proliferation, and invasion of VSMCs.^9^, ^61^ MFG‐E8 directly mediates the initial inflammatory responses in aged arteries or VSMCs.^62^ Recombinant MFG‐E8 (rMFG‐E8) was administered to the injured artery to accentuate the effect on age‐related vascular thickening and inflammation.^62^ Endogenous MFG‐E8 expression in aged common carotid arteries (CCA) is significantly induced by ligation injury.^62^ Aged CCAs treated with rMFG‐E8 exhibited increased leukocyte extravasation, cellular adhesion molecules expression, and increased NF‐ĸB activation in the ligated vessels.^62^ Treating early‐passage VSMCs from aged aortae with rMFG‐E8 substantially increased NF‐ĸB activation, proinflammatory gene expression, and cell proliferation.^62^ Notably, deletion of MFG‐E8 inhibited VSMC proliferation and neointimal formation upon damage, which became less in collagen deposition.^63^

### 5.6 NADPH oxidase

NADPH oxidase is a flavocytochrome b heterodimer, including two protein subunits, p22‐phox and either p91‐phox in fibroblasts or Nox1/2 in VSMCs. Aging increases Ang II^64^, ^65^, ^66^ and NADPH oxidase in VSMCs.^67^ Ang II increases NADPH oxidation of NOX‐2, which enhances MMP‐2 activation in VSMCs.^68^, ^69^, ^70^, ^71^ MMP‐2 increases TGF‐β1 and collagen production in aging VSMCs.^51^

### 5.7 Connective tissue growth factor

CTGF signaling plays a direct role in collagen production in VSMCs. CTGF, a cysteine‐rich protein, induced by TGF‐β1, is shown to trigger many cellular processes underlying fibrosis. In VSMCs, an activation pathway for collagen expression involving CTGF is induced by TGF‐β1.^22^ Treatment of VSMCs with CTGF results in increased collagen protein synthesis.^22^ These results suggest that TGF‐β1 can stimulate collagen synthesis by inducing CTGF, which is a downstream mediator of TGF‐β1.

### 5.8 Endothelin‐1

ET‐1 alters the synthesis of collagen in VSMCs.^72^, ^73^ ET‐1 is a strong stimulator of collagen type I, and a weak stimulator of collagen type III synthesis in VSMC.^73^ The pathway of ET‐1‐induced collagen production is not established and may be associated with TGF‐β1 activity.^72^

### 5.9 Nitric oxide

Nitric oxide (NO) has been implicated as an anti‐fibrotic agent. In vitro endothelial nitrogen oxygen synthase (eNOS) transferred into VSMC was shown to reduce the expression of MMP‐2 and MMP‐9 activity.^74^ TGF‐β1 activity was significantly reduced in VSMCs with over‐expression of eNOS, contributing to arterial calcification.^75^ Thus, reduced NO expression with aging may contribute to some of the alterations of TGF‐β1 in pathologic conditions in the vasculature and is potentially responsible for alterations in collagen remodeling. Aging aggravates nitrate‐mediated reactive oxygen species (ROS) and reactive nitrogen species (RNS) changes,^71^ likely promoting the process of arterial fibrosis.^76^

### 5.10 Vasorin

Ang II amplifies TGF‐β1 activation in the aged VSMCs of the arterial wall.^8^ Vasorin mRNA and protein expression were significantly decreased both in aortic walls and VSMCs in aging rats.^8^ Vasorin physically interacts with TGF‐β1 and functionally mitigates its fibrogenic signaling in VSMCs of the arterial wall.^8^ Treating young VSMCs with Ang II reduced vasorin protein expression to the levels of old untreated cells while treating old VSMCs with the Ang II AT1 receptor antagonist, losartan, upregulated vasorin protein expression up to the levels of young.^8^ Further, treating young VSMCs with Ang II increased the levels of MMP‐2 activation and TGF‐β1 downstream molecules, p‐SMAD‐2/3, and collagen type I production up to the levels of old untreated VSMCs, and these effects were substantially inhibited by overexpressing vasorin. Reduced expression of vasorin plays a contributory role in magnifying Ang‐II‐associated fibrogenic signaling in aged VSMCs of the arterial wall.^8^ Indeed, a chronic regimen of Ang II to young rats reduces the expression of vasorin and increases arterial fibrosis.^8^

In addition, vasorin also has effects on osteo‐/chondrogenic transdifferentiation and calcification of VSMCs.^7^ Vasorin inhibited the activation of the TGF‐β1‐induced pathway, SMAD2 phosphorylation, and downstream target gene expression and osteo‐/chondrogenic transdifferentiation of human VSMCs.^7^ Vasorin suppresses TGF‐β1 signaling and protects against osteo‐/chondrogenic transdifferentiation and calcification of VSMCs, reducing pro‐calcifying conditions.^7^

## 6 COLLAGEN PROMOTES PROINFLAMMATORY PHENOTYPIC SHIFTS OF VASCULAR CELLS

Collagen modification and degeneration affects the phenotypes of vascular cells, creating a fertile storm that potentially promote arterial proinflammation and stiffening (Figure 1).

### 6.1 Endothelial cells

Aging ECs are altered by modifications in deposition of collagen. Aged ECs undergo morphological changes that resemble the VSMC phenotype and express α‐smooth muscle actin (α‐SMA) and collagen type I.^77^ ECs are mechanosensitive to collagen stiffness. An increased intimal stiffness promotes endothelial dysfunction, such as a decrease in NO production and increased permeability of the physical barrier.^78^

### 6.2 Vascular smooth muscle cells

Young adult medial VSMCs placed in culture lose their contractility and their contractile myofilaments with passaging.^79^ They develop an extensive rough ER and a large Golgi complex, features often seen in old cells, which is accompanied by changes in myosin heavy chain isoforms smooth muscle myosin alpha 1 and 2 (SM1 and SM2) and upregulation of “synthetic” phenotype markers, such as inflammatory mediator MCP‐1; and the embryonic form of smooth muscle myosin heavy chain (SMemb), myosin light chain kinase (MLCK)‐210kD, and caldesmon.^26^, ^57^, ^80^, ^81^, ^82^, ^83^ In contrast, modulation into this “synthetic” phenotype can be inhibited by growing VSMCs on an ECM surface.^84^ Many of the phenotypic markers of VSMCs grown on fibrillar collagen follow more closely the phenotype of young adult medial VSMCs.^85^ Furthermore, culture of VSMCs on rigid gels of type IV collagen reproduces an even more closely phenotype of young medial VSMCs.^30^ In contrast, VSMCs cultured on monomer collagen retain many of the characteristics of synthetic VSMCs in developing lesions.^86^, ^87^

### 6.3 Migration and invasion

Invasion of the subendothelial space by VSMCs contributes to arterial wall aging. VSMCs embedded in a three‐dimensional collagen matrix form actin‐ and contactin‐rich extensions that enable to penetrate through holes in the matrix.^88^ ECM‐degrading actin‐rich protrusions are morphologically like the invadopodia formed by highly invasive metastatic tumor cells.^88^ VSMCs are durotactic, preferentially migrating in the direction of increased substrate stiffness, but their maximum migration speed depends on both ECM stiffness and ECM chemical cues.^89^, ^90^, ^91^ Interestingly, increased ECM stiffness enhances VSMC migration induced by platelet derived growth factor (PDGF) signaling.^92^

### 6.4 Proliferation

Fibrillar collagen inhibits VSMC proliferation through the regulation of cell cycle inhibitors. Degraded collagen may be required to release VSMCs from non‐permissive states. It is not just the type of matrix protein that VSMCs are in contact with that influences growth and differentiation properties but also the organization and presentation of specific matrix components.^93^ VSMCs adhering to native fibrillar collagen did not undergo mitogenesis when stimulated with PDGF.^87^ However, VSMCs adhering to denatured collagen responded to PDGF and underwent cell division.^87^ Degraded collagen fragments cause VSMCs to lose their focal adhesion and to round up, and are ready to migrate or proliferate.^94^ Furthermore, it has been observed that VSMCs in aortic rings of a normal vessel cultured in a physiologic bath will not proliferate in response to PDGF.^95^ However, if the same aortic rings are briefly treated with a combination of collagenase and elastase and then placed in the same bath, the VSMCs in the aortic ring will proliferate in response to exogenously added PDGF.^95^ Similar to migration, VSMC proliferation is also dependent on ECM ligand density, but the increase in matrix stiffness is the dominant factor affecting proliferation.^96^ Interestingly, increased matrix stiffness enhances VSMC proliferation induced by PDGF.^92^

### 6.5 Stiffening

Individual VSMCs derived from aged animals show more stiffening than those derived from the young adult animals over a wide frequency range of the imposed oscillatory deformation.^97^ Notably, profibrotic TGF‐β1 emerges as a specific modifier of age‐associated VSMCs stiffening in vitro.^97^ TGF‐β1 reinforces the mechanical phenotype of arterial aging in VSMCs on multiple time and length scales through the clustering of mechanosensitive integrins, α_5_β_1_ and α_v_β_3_.^97^ Thus, an intervention of the long‐range increase of VSMCs stiffness via fibrotic TGF‐β1 signaling with aging is a useful therapeutic approach to mitigate arterial wall stiffening.

## 7 INTERVENTIONS OF ARTERIAL PROFIBROSIS WITH AGING

### 7.1 Physical exercise

Regular aerobic exercise reduces large elastic arterial stiffening and optimizes arterial compliance and preserves endothelial function.^39^ Thus, regular aerobic exercise is viewed as a “first line” strategy for the prevention and treatment of arterial aging. Habitual aerobic exercise decreases MMP‐2, TGF‐β1, and collagen production in the arterial wall.^39^ Even moderate‐intensity exercise training attenuates aortic stiffening accompanied by reduced collagen levels and prevents endothelial dysfunction, marked by the restoration of endothelium‐mediated vascular relaxation of aged rat aortae in response to acetylcholine.^98^ Notably, exercise training also reduced the ratio of collagen to elastin in old rat arteries, contributing to a reduction of stiffening and an increase of compliance during arterial aging.^99^

### 7.2 Calorie restriction

Multiple health benefits of calorie restriction (CR) are widely recognized. Long‐term daily CR delays age‐associated changes in the cardiovascular system by reducing the rate of collagen deposition.^100^ CR also protects against the age‐associated increase of transcription factors p‐c‐Jun and p‐38 kinase activation, involved in TGF‐β1 expression, activation, and signaling, contributing to arterial fibrosis.^101^ A recent study shows that CR markedly reduces age‐associated IMT, collagen deposition, and elastin fractionation/degradation within the arterial walls in rats.^83^ CR effectively prevents an age‐associated increase in MMP‐2, and TGF‐β1, and its downstream signaling molecule, p‐SMAD‐2/3, a profibrogenic signaling pathway in the arterial wall in aging rats. Interestingly, in early‐passage cultured VSMCs isolated from ad libitum (AL) and CR rat aortae, CR alleviates the age‐associated VSMC phenotypic shifts, profibrogenic signaling, and migration/proliferation in response to PDGF.^83^ Taken together, CR reduces the matrix and cellular profibrosis that occurs within the aging aortic wall.

### 7.3 Renin angiotensin system blockade

Rats that received a chronic regimen of the ACE‐1 inhibitor, enalapril, which demonstrated substantial impedance of arterial aging, showed a decrease in collagen deposition.^102^, ^103^ Furthermore, the ACE inhibitor, enalapril, and the AT1 antagonist, losartan, effectively prevent not only collagen deposition but also the degradation of the aging internal elastic lamina.^104^

### 7.4 MMP inhibition

MMP inhibition is broadly subdivided into non‐synthetic, such as endogenous tissue inhibitor of metalloproteinases (TIMPs), and synthetic small molecules. MMP inhibition via synthetic inhibitor PD166793 retards TGF‐β1 activation, SMAD signaling, collagen production, and elastolysis. It also increases vasorin protein, contributing to the prevention of aging‐related arterial fibrosis, elastolysis, calcification, and blood pressure increase.^6^, ^7^, ^8^

### 7.5 Senolysis

Collagen fibrils become resistant to cleavage over time. Mice with a targeted mutation (Col1a1(r/r)) that yields collagenase‐resistant type I collagen create an old senescent phenotype. VSMCs in the aortic wall of these mutated mice are susceptible to stress‐induced senescence, displaying senescence‐associated beta‐galactosidase (SA‐β‐Gal) activity and upregulated p16 in response to Ang II infusion.^105^ In addition, mutant collagen directly reduces the replicative lifespan of human VSMCs and increases stress‐induced SA‐β‐Gal activity, p16 expression, and p21 expression.^105^ Thus, resistance to collagen cleavage, such as AGE formation, accelerates cellular senescence, arterial stiffening, and aging. A long‐term senolytic treatment (intermittently with dasatinib + quercetin via oral gavage) significantly eliminates senescent cells in the medial layer of aortae from aged mice and reduces arterial stiffening.^106^

## 8 CONCLUDING REMARKS

Proinflammation coupled with profibrotic signaling leads to arterial fibrosis. Thus, reducing proinflammation or profibrotic conditions should reduce the incidence of  fibrogenic associated adverse vascular events, such as IMT, that facilitate the development of hypertension and atherosclerosis. Steadfast regulation of collagen is critical to the homeostasis of the arterial wall. Vascular collagen is not only a major component of the scaffold for vascular cells but also affects the phenotype of the ECs and VSMCs. A malfunction in any of the collagen‐regulatory steps (synthesis, secretion, or degradation) can potentially lead to abnormal structure and dysfunction of the blood vessel. Age increases the prevalence of arterial stiffening since the thickened wall becomes rich with collagen deposition. Age‐associated collagen deposition is regulated and modified by proinflammatory molecules, such as renin angiotensin system (RAS), TGF‐β1, MMP, ROS, and NO. Adverse collagen remodeling accelerates arterial stiffening in the elderly by further altering the phenotype of vascular cells, creating the fertile soil for the pathogenesis of hypertension and atherosclerosis. Thus, preventing profibrosis through the manipulation of collagen deposition and alleviating proinflammation is a novel approach to preventing age‐associated stiffening conditions, such as hypertension and atherosclerosis.

## CONFLICTS OF INTEREST

Nothing to disclose.

## AUTHOR CONTRIBUTIONS

M. W.: Conceived and designed this work, reviewed the literature, and wrote the manuscript. R. E. M.: Revised and critically edited the manuscript. K. R. M.: Created a thematic diagram, edited and critically revised the manuscript.
